# Early treatment monitoring of multidrug-resistant tuberculosis based on CT radiomics of cavity and cavity periphery

**DOI:** 10.1186/s41747-025-00581-2

**Published:** 2025-04-26

**Authors:** Xinna Lv, Ye Li, Chenyu Ding, Lixin Qin, Xiaoyue Xu, Ziwei Zheng, Dailun Hou

**Affiliations:** 1 Department of Radiology, Beijing Chest Hospital, Capital Medical University, Beijing, China; 2 Department of Radiology, Shandong Provincial Hospital Affiliated to Shandong First Medical University, Jinan, China; 3 Department of Radiology, Wuhan Pulmonary Hospital, Wuhan, China

**Keywords:** Radiomics, Sputum, Tomography (x-ray computed), Treatment failure, Tuberculosis (multidrug-resistant)

## Abstract

**Background:**

Early identification of treatment failure can effectively improve the success rate of antituberculosis treatment. This study aimed to construct a predictive model using radiomics based on cavity and cavity periphery to monitor the early treatment efficacy in multidrug-resistant tuberculosis (MDR-TB).

**Methods:**

We retrospectively collected data on 350 MDR-TB patients who underwent pretreatment chest computed tomography (CT) and received longer regimens from two hospitals. They were subdivided into training (252 patients from hospital 1) and testing (98 patients from hospital 2) cohorts. According to at least two consecutive sputum culture results within the early sixth months of treatment, patients were divided into high-risk and low-risk groups. Radiomics models were established based on cavity and periphery with a range of 2, 4, 6, 8, and 10 mm. A combined model fused radiomics features of cavity with the best-performing peripheral regions. The performance of these models was evaluated by the receiver operating characteristic area under the curve (AUC) and clinical decision curve analysis.

**Results:**

The cavity model achieved AUCs of 0.858 and 0.809 in the training and testing cohort, respectively. The radiomics model based on 4 mm peripheral region showed superior performance compared to other surrounding areas with AUCs of 0.884 and 0.869 in the two cohorts. The AUCs of the combined model were 0.936 and 0.885 in the two cohorts.

**Conclusion:**

CT radiomics analysis integrating cavity and cavity periphery provided value in identifying MDR-TB patients at high risk of treatment failure. The optimal periphery extent was 4 mm.

**Relevance statement:**

The cavity periphery also contains therapy-related information. Radiomics model based on cavity and 4 mm periphery is an effective adjunct to monitor early treatment efficacy for MDR-TB patients that can guide clinical decision.

**Key Points:**

- A combined CT radiomics model integrating cavity with periphery can effectively monitor treatment response.
- A periphery of 4 mm showed superior performance compared to other peripheral smaller or greater extent.
- This study provided a surrogate for identifying the high risk of treatment failure in multidrug-resistant tuberculosis patients.

**Graphical Abstract:**

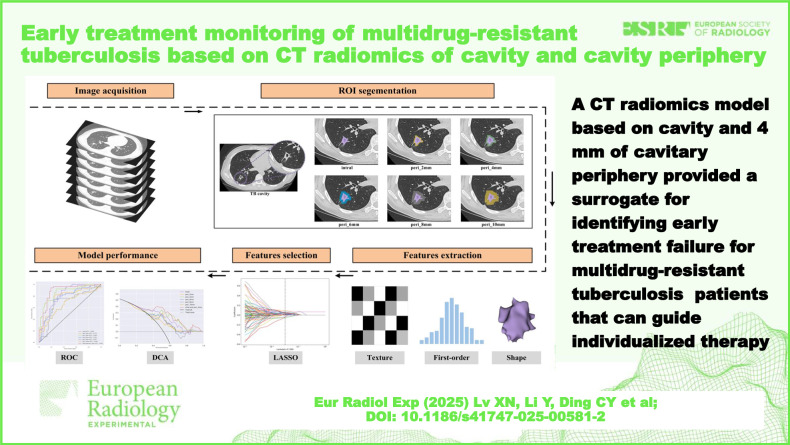

## Background

Tuberculosis (TB), caused by *Mycobacterium tuberculosis*, continues to pose a significant public health crisis worldwide [1]. Recently, the burden of drug-resistant TB has risen, with multidrug-resistant tuberculosis (MDR-TB) emerging as a primary concern for TB control [2, 3]. Longer MDR-TB regimens are recommended for MDR-TB in World Health Organization guidelines [3, 4]. This strategy involves complex management due to the required combination of multiple antituberculosis drugs and longer duration [5, 6]. Successful treatment hinges on closely monitoring treatment response and implementing individualized management strategies. Evaluating bacteriological conversion status during therapy is crucial for assessing TB treatment response [3, 7, 8]. However, bacterial contamination and irregular sampling can affect sputum quality and then reduce the predictive sensitivity [9]. Additionally, sputum culture results take 4–8 weeks, potentially delaying treatment initiation [10].

Chest CT is a routine technique used to diagnose and monitor pulmonary TB. Cavitation, resulting from the expulsion of necrotic tissue through the bronchial tree, is a classic indicator of MDR-TB [11, 12]. Evidence has demonstrated that cavitation is associated with high mycobacterial burden and poor treatment outcomes [11]. Detailed quantitative analysis of MDR-TB cavities could serve as an effective adjunct to assess the treatment response. Radiomics converts medical images into high-dimensional quantitative features and provides significant insights into the pathophysiology of lesion. Previous studies have explored the potential of CT radiomics to predict MDR-TB [10, 13], including one study that developed a cavity-based radiomics to predict the sputum culture conversion at the sixth month for MDR-TB [7]. Histological analysis reveals a region of granulomatous pneumonia at the boundary between the cavity wall and healthy tissue, filled with inflammatory cells [11]. This region is involved in the immune response and may influence treatment outcomes. Although CT radiomics signature based on the peritumoral region has shown promise in predicting therapy efficacy in breast and cervical cancer [14, 15], no studies have yet investigated the additional value of the cavitary periphery in treating MDR-TB.

This study aimed to assess the predictive utility of CT radiomics features based on cavity and cavity periphery in monitoring early treatment efficacy in MDR-TB patients on longer regimens, and further explore the most effective region for prediction.

## Methods

### Study population

This retrospective study was approved by the ethics committees of Beijing Chest Hospital, Capital Medical University (No. 36, 2021) and Wuhan Pulmonary Hospital, and the requirement for informed consent was waived.

All enrolled patients in our study met the following inclusion criteria: (1) confirmed diagnosis of active TB through sputum culture, microscopy, or polymerase chain reaction test; (2) determination of MDR-TB by drug susceptibility testing; (3) at least two results of sputum culture within early 6 months of treatment on longer regimens; (4) CT images obtained prior to treatment; and (5) at least a cavitary lesion in the CT scan. Patients were excluded based on the following criteria: (1) history of other pulmonary diseases, human immunodeficiency virus infection or diabetes; (2) irregular or interrupted treatment; and (3) incomplete imaging or clinical information and existence of image artifacts.

According to at least two consecutive sputum culture results within the early sixth months of treatment, patients with at least two consecutive positive results or more were defined as high-risk groups, while the others were defined as low-risk groups.

**Fig. 1:**
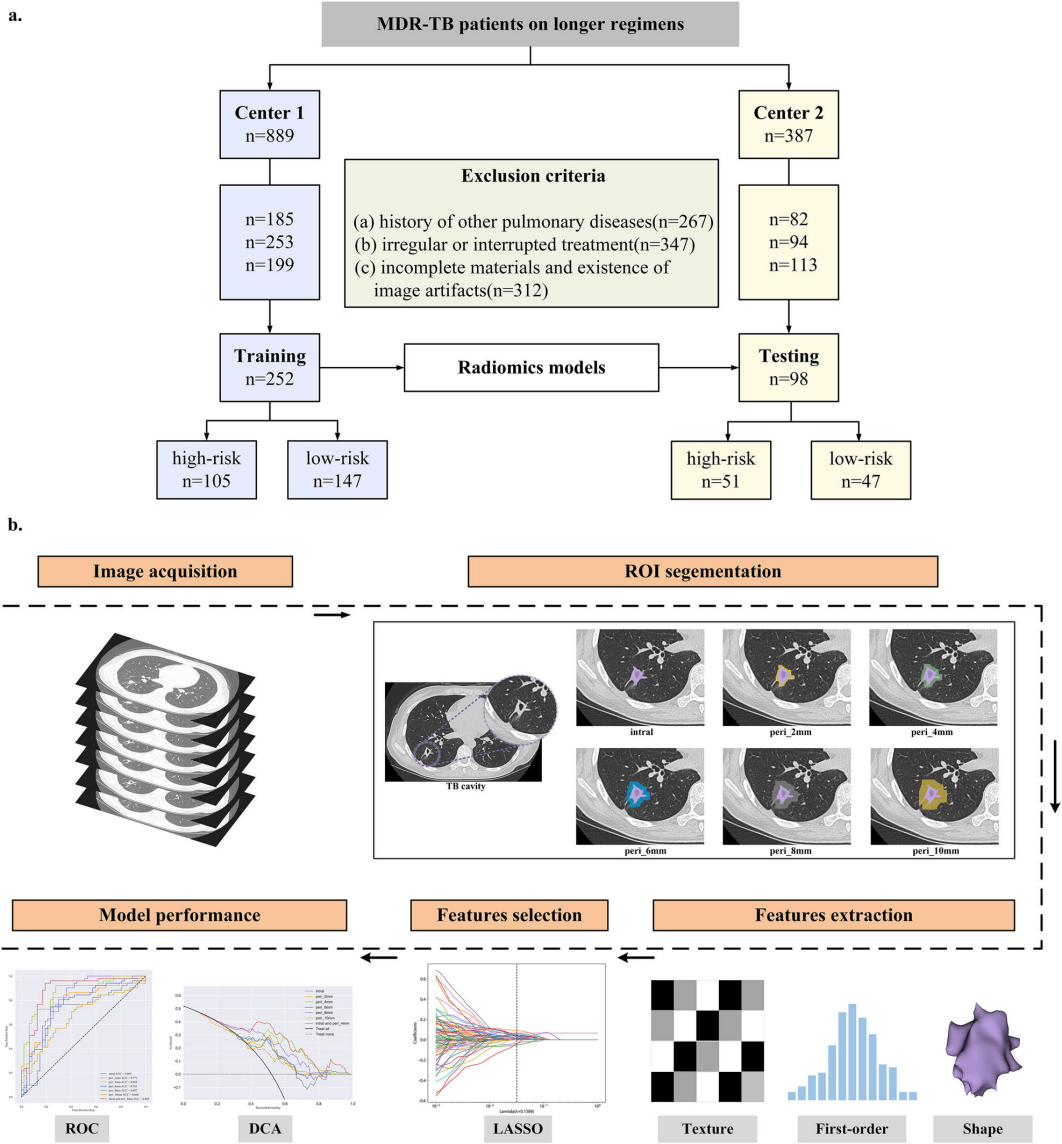
**a** Flowchart of patients enrollment and exclusion criteria. **b** Workflow of radiomics analysis. Radiomics features were extracted from cavity and regions at the periphery of the cavity of 2, 4, 6, 8, and 10 mm. For feature selection, least absolute shrinkage and selection operator (LASSO) regression was used. The predictive performance of radiomics model was assessed by area under the receiver operating characteristic curve and decision curve analysis

Finally, a total of 350 MDR-TB patients were enrolled. Among them, 252 MDR-TB patients retrospectively recruited from hospital 1 between March 2014 and October 2023 composed the training cohort and 98 patients recruited from hospital 2 between August 2020 and December 2023 composed the testing cohort. The patient recruitment flowchart is shown in Fig. 1a.

### CT examination

All patients underwent a pretreatment CT scan using a Light Speed VCT, Revolution CT, or Optima CT 680 system (GE Healthcare, Waukesha, WI, USA). Scanning parameters are presented in detail in the Supplementary material.

### Segmentation and processing

First, the internal regions of interest (ROIs) were delineated slice by slice for the cavity using “3D slicer” (http://www.slicer.org) by a radiologist with 5 years of experience in lung CT and then confirmed or modified by a radiologist with 10 years of experience in lung CT. The two radiologists were blind to the consecutive sputum culture results of MDR-TB patients. In patients with multiple cavities, only the largest one was used for the analysis of radiomics.

Second, CT images were resampled to the same voxels of 1 × 1 × 1 mm^3^ to reduce the effect of the slice thickness variation. Then, the region at the periphery of the cavity was obtained using Python (3.9, Python Software Foundation, Amsterdam, The Netherlands) to automatically expand ROIs. Finally, we created 6 ROIs which included the internal ROI, 2-mm peripheral ROI (peri_2mm), 4-mm peripheral ROI (peri_4mm), 6-mm peripheral ROI (peri_6mm), 8-mm peripheral ROI (peri_8mm), and 10-mm peripheral ROI (peri_10mm). The process of ROI expansion is shown in the Supplementary material.

### Feature extraction

To normalize the intensity of the image signal and reduce variations in scanner characteristics, grayscale discretization of the ROIs was set by a fixed bin width of 25 HU. In addition, we used the Laplacian of Gaussian filter, Gradient filter, Wavelet filter, Square filter, Square Root filter, Logarithm filter, and Exponential filter to preprocess the original images.

The radiomics features that were confirmed to be international Biomarker Standardization Initiative compliant were extracted using Pyradiomics package in Python. Then the z-score transformation was applied to standardize the radiomics features. We extracted radiomics features from these 6 ROIs. Finally, a total of 1,835 radiomics features were extracted from each ROI based on the original and filtered images, which contained first-order features, shape and size features, and texture features. Detailed information of the radiomics features is available in the PyRadiomics official documentation (https://pyradiomics.readthedocs.io/en/latest/features.html).

### Feature selection and model construction

To select the most important features of prediction, we took many steps, including independent sample *t*-test, Pearson correlation analysis and the least absolute shrinkage and selection operator. The feature selection process is provided in the Supplementary material.

The optimal selected radiomics features were used to establish 6 radiomics models respectively. Then, we establish a combined model combing the cavity and the optimal periphery ROI, which was selected by the best performance of five periphery ROIs in prediction. The multilayer perceptron was used to construct these radiomics models. These models were all trained and validated on the training cohort, which was randomly divided into parts at a ratio of 7:3 using tenfold cross-validation. After that, the best model was selected and tested in the testing cohort. The process of feature selection and model construction was carried out by the Python Scikit-learn package (version 3.8, Scikit-learn Version 0. 21, http://scikit-learn.org/).

### Statistical analysis

Statistical analysis was performed using SPSS software (version 26) and Python Scikit-learn package. The independent *t*-test and the χ^2^ test were used to assess statistical differences in continuous and categorical variables across two cohorts, respectively. The predictive performance of these models was evaluated using the area under the receiver operator characteristic curve (AUC), accuracy, precision (positive predictive value), recall (sensitivity), and F1 score in both the training and testing cohorts. The receiver operating characteristic curve was plotted, and the 95% confidence interval (CI) was calculated. The decision curve analysis was used to evaluate the clinical usefulness of these models by calculating the net benefits at different threshold probabilities in two cohorts. Values of *p* lower than 0.050 indicated a significant difference. Figure 1b shows the workflow of this study.

## Results

### Clinical characteristics of patients

Table 1 shows the basic clinical characteristics of all MDR-TB patients. No significant difference was found between the two groups in terms of gender, alcohol consumption, and smoking in both training and testing cohorts. As for the age, it only has statistical significance in the training cohort (*p* = 0.019), while has no significant difference in the testing cohort (*p* = 0.098).

**Table 1:** Clinical characteristics of the MDR-TB patients in the training and testing cohorts

Characteristics	Training cohort (*n* = 252)		*p*-value	Testing cohort (*n* = 98)		*p*-value
	High-risk	Low-risk		High-risk	Low-risk	
	(*n* = 105)	(*n* = 147)		(*n* = 51)	(*n* = 47)	
Age, years (mean ± standard deviation)	41.6 ± 13.3	37.4 ± 14.4	0.019*	40.4 ± 11.9	35.9 ± 14.7	0.098
Gender, *n* (%)						
Male	74 (70.5)	92 (62.6)	0.193	36 (70.6)	40 (85.1)	0.085
Female	31 (29.5)	55 (37.4)		15 (29.4)	7 (14.9)	
Alcohol consumption, *n* (%)						
Yes	19 (18.1)	40 (27.1)	0.092	22 (43.1)	22 (46.8)	0.715
No	86 (81.9)	107 (72.9)		29 (56.9)	25 (53.2)	
Smoking, *n* (%)						
Yes	46 (43.8)	53 (35.9)	0.214	23 (45.1)	16 (34.0)	0.264
No	59 (56.2)	94 (64.1)		28 (54.9)	31 (66.0)	

### Radiomics feature selection

**Fig. 2:**
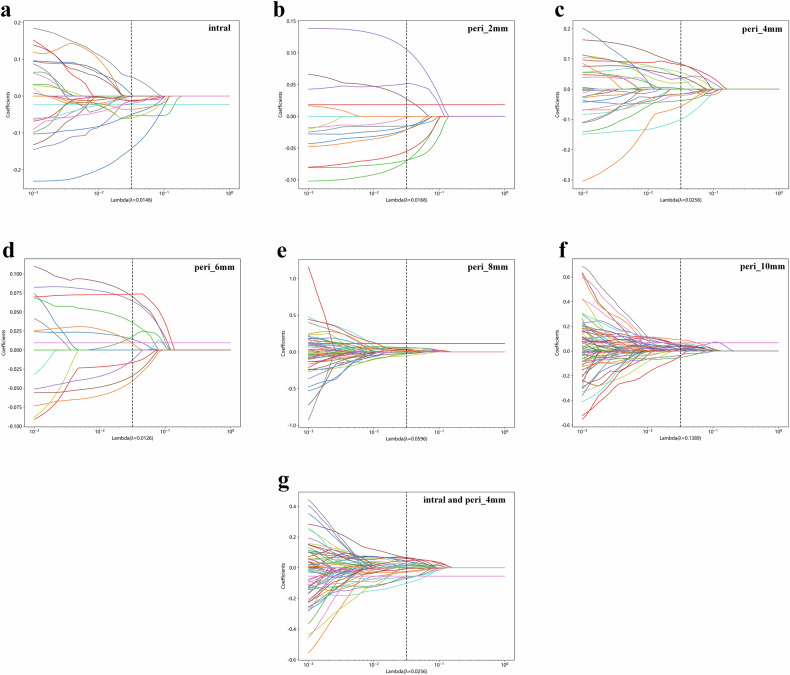
The least absolute shrinkage and selection operator coefficients *versus* log (Lambda) of seven models: (**a**) cavity, (**b**) peri_2mm, (**c**) peri_4mm, (**d**) peri_6mm, (**e**) peri_8mm, (**f**) peri_10mm, and (**g**) combined models

**Fig. 3:**
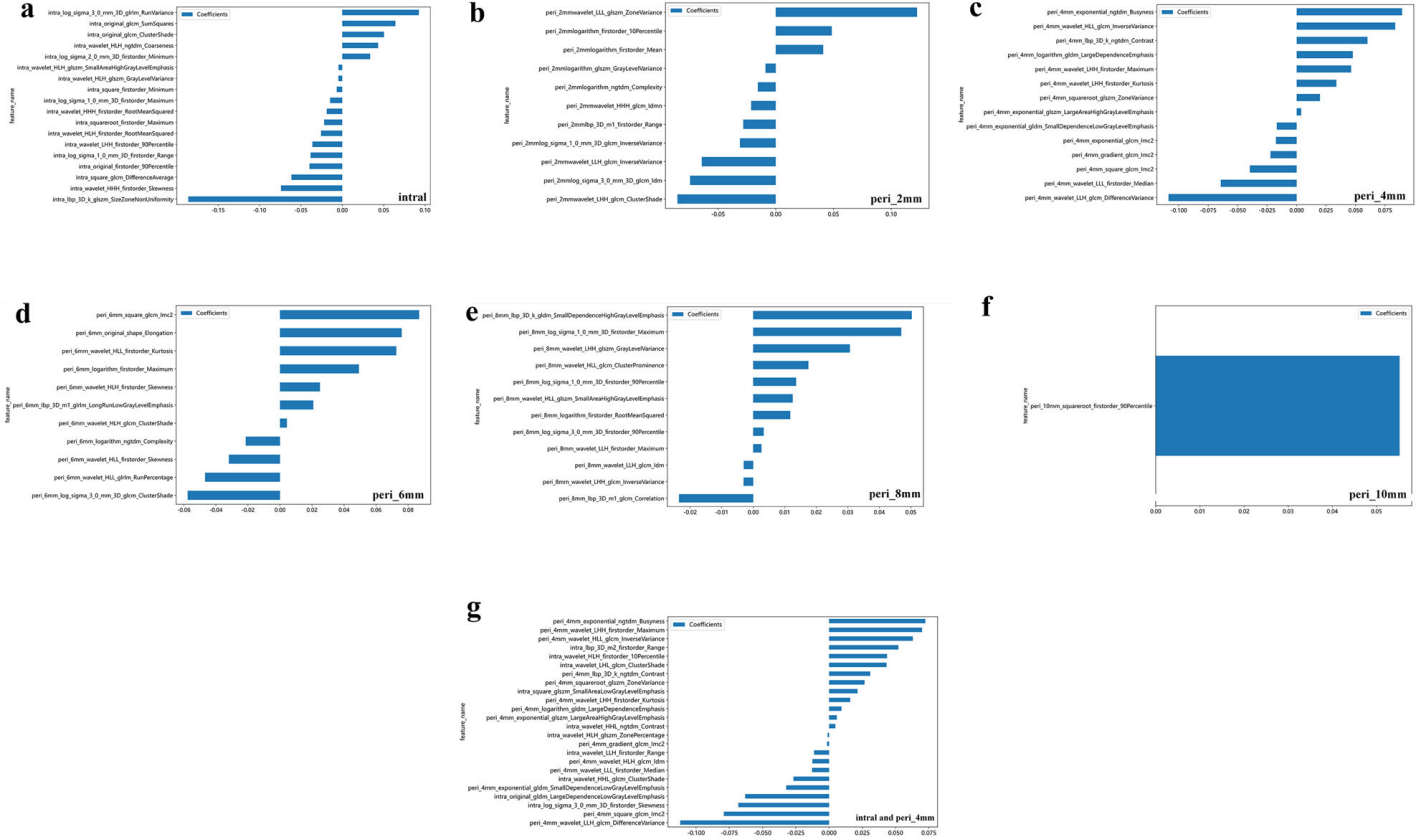
The normalized importance of radiomics features in the seven models. The 18, 11, 14, 11, 12 and 1 radiomics features based on (**a**) cavity, (**b**) peri_2mm, (**c**) peri_4mm, (**d**) peri_6mm, (**e**) peri_8mm, (**f**) peri_10mm, and (**g**) combined models with the highest normalized importance, respectively, were selected and included

This study extracted 1,835 features based on the cavity and five regions at the periphery of the cavity separately. Through independent sample *t*-test, Pearson correlation analysis and the least absolute shrinkage and selection operator for feature selection, we selected 18, 11, 14, 11, 12, and 1 features from the six ROIs, respectively, which were potentially related to early treatment failure risk. The least absolute shrinkage and selection operator coefficients *versus* log (Lambda) are shown in Fig. 2, and the normalized importance of these features for each model is shown in Fig. 3.

### Performance of prediction models

**Fig. 4:**
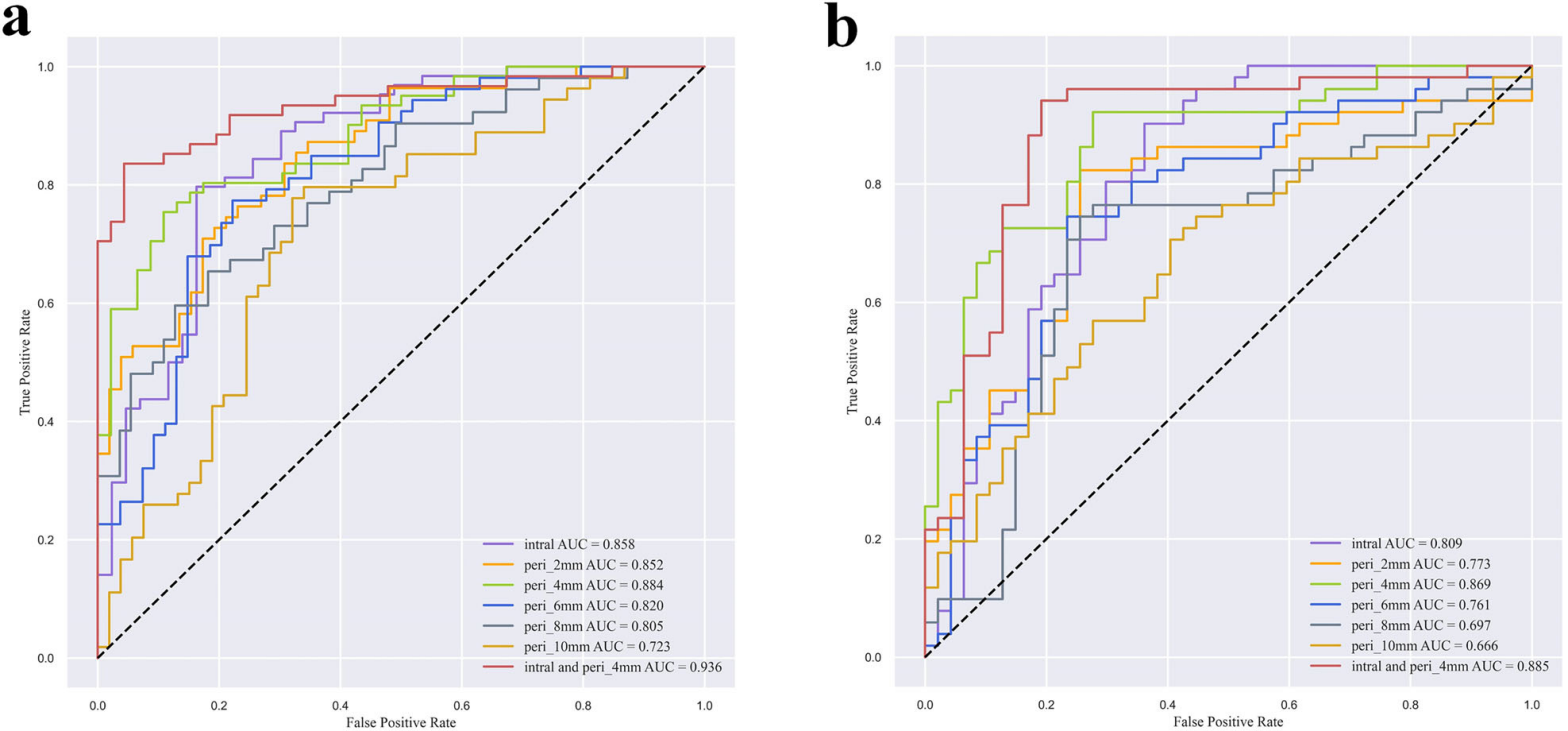
Receiver operating characteristic curves of the models. **a** Training cohort; **b** Testing cohort

The AUC, accuracy, sensitivity, specificity, and F1 score of each model in the two cohorts are presented in Table 2. The cavity radiomics model showed AUCs of 0.858 (95% CI 0.780–0.931) and 0.809 (95% CI 0.718–0.893) in the training and testing cohort, respectively. The radiomics model of peri_2mm achieved the AUCs of 0.852 (95% CI 0.783–0.922) and 0.773 (95% CI 0.669–0.868) in two cohorts. The performance of peri_4mm model was slightly higher than cavity radiomics model and much higher than other cavitary periphery models with AUCs of 0.884 (95% CI 0.823–0.945) in the training cohort, 0.869 (95% CI 0.797–0.930) in the testing cohort. The AUCs of peri_6mm model were 0.820 (95% CI 0.741–0.900) and 0.761 (95% CI 0.648–0.853) in the training and testing cohort, respectively. The peri_8mm model separately yielded AUCs of 0.805 (95% CI 0.725–0.886) and 0.697 (95% CI 0.592–0.804) in two cohorts. The radiomics model of peri_10mm performed the worst. The AUCs were 0.723 (95% CI 0.625–0.821) in the training cohort and 0.666 (95% CI 0.553–0.778) in the testing cohort. We fused the radiomics features of cavity and peri_4mm to establish a combined model. Finally, the combined model including 10 radiomics features based on cavity and 14 features based on peri_4mm achieved the best performance with the AUC of 0.936 (95% CI 0.891–0.981) in the training, 0.885 (95% CI 0.809–0.953) in the testing cohort. The receiver operating characteristic curve of each model is shown in Fig. 4.

**Table 2:** Predictive performance of different models in the training and testing cohorts

Index	Training cohort							Testing cohort						
	Intra- cavity	peri_2mm	peri_4mm	peri_6mm	peri_8mm	peri_10mm	Intra-cavity with peri_4mm	Intra-cavity	peri_2mm	peri_4mm	peri_6mm	peri_8mm	peri_10mm	Intra-cavity with peri_4mm
AUC	0.858	0.852	0.884	0.820	0.805	0.723	0.936	0.809	0.773	0.869	0.761	0.697	0.666	0.885
Accuracy	0.804	0.757	0.804	0.766	0.729	0.720	0.879	0.776	0.786	0.827	0.755	0.745	0.653	0.878
Sensitivity	0.781	0.691	0.738	0.755	0.691	0.759	0.820	0.902	0.824	0.922	0.745	0.745	0.706	0.941
Specificity	0.837	0.827	0.891	0.778	0.769	0.679	0.957	0.638	0.745	0.723	0.766	0.745	0.596	0.809
Recall	0.781	0.691	0.738	0.755	0.691	0.759	0.820	0.902	0.824	0.922	0.745	0.745	0.706	0.941
F1	0.826	0.745	0.811	0.762	0.724	0.732	0.885	0.807	0.800	0.847	0.760	0.752	0.680	0.889

*AUC* Area under the receiver operating characteristic curve, *peri_2mm* 2-mm peripheral ROI, *peri_4mm* 4-mm peripheral ROI, *peri_6mm* 6-mm peripheral ROI, *peri_8mm* 8-mm peripheral ROI, *peri_10mm* 10-mm peripheral ROI, *ROI* Region of interest

**Fig. 5:**
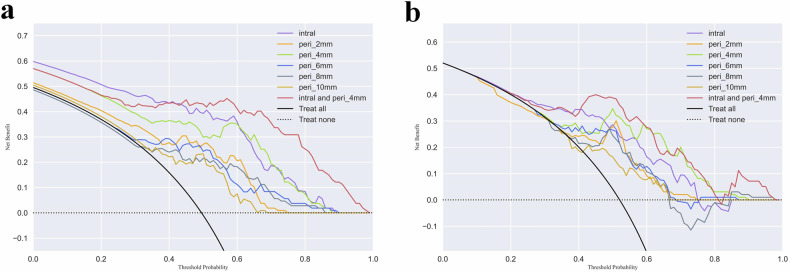
Decision curve analysis of the models. **a** Training cohort; **b** Testing cohorts

Decision curve analyses displayed the clinical practice value of the predictive models (Fig. 5). The combined model integrating internal cavity with peri_4mm region in this study added more clinical net benefit to predict treatment failure if the threshold probability of the patients is between 0.4 and 1.0 in both the training and testing cohorts.

## Discussion

This study assessed the response of MDR-TB patients receiving longer regimens within the first 6 months and identified those at high risk of treatment failure. A total of 350 MDR-TB patients from two centers were enrolled and stratified into high-risk and low-risk groups. Radiomics features were extracted from cavity and cavity periphery of 2-, 4-, 6-, and 10-mm range using pretreatment CT images. We found that the features combined with internal cavity and 4-mm peripheral region exhibited optimal performance, serving as a potential predictor for monitoring treatment response in these patients.

Almost 70% of MDR-TB patients exhibit cavity, which is linked to treatment failure and recurrence [12, 16, 17]. Variations in cavity size can indicate the therapeutic efficacy of MDR-TB treatment [13]. If the cavity does not diminish in size during treatment, it is essential to adjust therapy strategy or even consider surgical intervention to eliminate persistent sources of infection [12, 13]. Closely monitoring cavity changes during treatment is significant to evaluate the treatment response of MDR-TB. A granulomatous pneumonia region can be found at the border between the healthy lung tissue and the cavity wall, where the lung tissue structure is intact, but the alveoli are filled with inflammatory cells [11, 16]. Cavity formation primarily involves the transition from an immune-accessible region to an immune-sheltered surface [11, 18]. Both innate and adaptive immune responses play a role in the formation and development of cavity [19]. Therefore, the granulomatous pneumonic region around the cavity may provide therapeutically relevant information.

Radiomics has shown potential value in the diagnosis, clinical or pathological classification, severity and prognosis assessment of various diseases and, more recently, in the field of TB [7, 10, 13–15, 18, 20]. A previous study evaluated the predictive ability of CT radiomics in differentiating lung cancer from TB [20]. It found that chest CT radiomics can be considered a noninvasive tool to differentiate the two diseases. Zou et al [21] recruited 204 drug-resistant TB patients who completed treatment and collected their two follow-up CT images. Three radiomics signature-based models, two deep learning models and a fused model were built to predict early treatment outcomes. The combination of radiomics features with deep learning model achieved the best performance to detect early failure of drug-resistant TB. Despite these researches demonstrating the effectiveness of radiomics in TB diagnosis and therapeutic assessment, a more precise quantitative analysis of TB, in particular the MDR-TB, remained unavailable.

Given the diverse imaging findings and substantial individual differences in MDR-TB patients, a more precise and targeted approach was needed. A cavity-based radiomics model had been previously constructed to predict the sputum culture conversion at the sixth month for MDR-TB patients, with a model only reflecting the sputum culture status at the sixth month, which may delay the detection of treatment failure. In recent years, the value of peritumoral radiomics features has been widely discussed for various cancers [15, 22]. Mao et al [15] confirmed that the intratumoral and peritumoral radiomics performed well in preoperatively predicting the effect of neoadjuvant chemotherapy of breast cancers. Similarly, Hu et al [22] found that a combination of peritumoral and intratumoral features can accurately assess the treatment outcomes in locally advanced esophageal squamous cell carcinoma. The region surrounding the TB cavity is filled with many immune cells, yet the impact of these regions on efficacy monitoring remains unexplored.

In our study, we extracted and selected 18 radiomics features from internal cavities and 11, 14, 11, 12, and 1 features from 2-, 4-, 6-, and 10-mm cavitary periphery regions separately. Based on these features, six predictive models were established to assess the early treatment response for MDR-TB patients. The peri_4mm radiomics model performed optimally, achieving an AUC of 0.869 in the testing cohort, with higher sensitivity, specificity, and F1 score compared to other periphery models. With an AUC value of 0.858 in the training and 0.809 in the testing cohort, the internal cavity model performed slightly worse than the peri_4mm model.

These findings align with previous studies [23, 24]. Cui et al [23] evaluated the power of intra-tumor and peritumor radiomics to distinguish lung cancer from TB. They revealed that the peritumoral area showed higher discriminative potential than the intratumoral area, especially the 4-mm area outside the lesion. Li et al [24] discussed whether the cavity region within nodules affects the predictive ability to identify lung adenocarcinoma and TB. According to their results, the exclusion of the cavitary area exerts no significant influence on the results. This result may be related to the fact that the major pathological components within the cavity are necrosis and destructed lung tissue.

Of note, our predictive power of the peri_2mm model performed lower than the peri_4mm model, and model efficiency gradually decreased with broader scope inclusion. This suggests that inflammatory cells primarily involved in cavity development are concentrated in the cavitary periphery 4-mm region. Thus, a combined model integrating internal cavity and peri_4mm radiomics was established. The combined model yielded an excellent performance (AUC of 0.885) in the testing cohort and added more clinical net benefit than the other models according to the decision curve analysis: combining cavity and cavitary periphery radiomics can effectively enhance predictive power to monitor early sputum culture.

This study has several limitations. First, the sample size is small. It is essential to collect patients from more centers and carry out prospective validation in the future. Second, the manual contouring of ROI segmentation is labor-intensive and time-consuming, highlighting the urgent need for the development of an automatic segmentation algorithm to facilitate lesion identification. Finally, other common imaging signs, such as nodules and tree-in-bud, also need to be quantitatively analyzed.

To conclude, this study demonstrated that the region at the periphery of the cavity, specifically the peripheral 4-mm region, holds the potential to effectively reflect the treatment response. The combined model based on internal cavity and cavitary periphery 4-mm CT radiomics provides an auxiliary tool to early monitor the sputum culture status for MDR-TB patients undergoing longer regimens, which aids in identifying patients at high risk of treatment failure and may enable timely adjustment of therapeutic strategies.

## Supplementary information

ELECTRONIC SUPPLEMENTARY MATERIAL

## Data Availability

The datasets used and/or analyzed during the current study are available from the corresponding author upon reasonable request.

## Abbreviations

AUC: Area under the receiver operator characteristic curve
CI: Confidence interval
CT: Computed tomography
MDR-TB: Multidrug-resistant tuberculosis
peri_10mm: 10-mm peripheral ROI
peri_2mm: 2-mm peripheral ROI
peri_4mm: 4-mm peripheral ROI
peri_6mm: 6-mm peripheral ROI
peri_8mm: 8-mm peripheral ROI
ROI: Region of interest
TB: Tuberculosis
