# Supplementary material for: Early treatment monitoring of multidrug-resistant tuberculosis based on CT radiomics of cavity and cavity periphery
Source: Eur Radiol Exp. 2025 Apr 26;9:43. doi: 10.1186/s41747-025-00581-2 (PMC12033146; doi:10.1186/s41747-025-00581-2)
Supplement: Supplementary file 1 — ELECTRONIC SUPPLEMENTARY MATERIAL [file 41747_2025_581_MOESM1_ESM.pdf]

Early treatment monitoring of multidrug-resistant tuberculosis based on CT radiomics of cavity and cavity periphery

ELECTRONIC SUPPLEMENTARY MATERIAL

Scanning parameters

The scanning parameters for the chest CT were as follows: tube voltage, 120 kVp; automatic tube current modulation; detector collimation, 64 × 0.625mm; rotation time, 500 ms; and pitch, 1.375. The image reconstruction parameters were as follows: slice thickness, 1.25 mm; increment, 1.25 mm; field of view, 15 cm; and matrix, 512 × 512.

Process of ROI expansion

The original ROI mask was systematically extended using the morphological dilation operator at varying radial distances. Different peritumoral regions were explored by configuring dilation intervals of 2, 4, 6, 8, and 10 mm to assess their impact on the predictive capabilities of the model. We used this method to enlarge the cavity mask by a specified value. The cavitary region was represented as a binary mask, where the cavities were marked as 1 and the peripheral regions were denoted as 2. The dilation of the cavity mask was then achieved using a spherical structuring element, corresponding to the desired extension distance. The regions outside the lung after dilation such as the chest wall and mediastinum were excluded for further analysis.

Feature selection

First, independent sample t-test initially selected significantly different features between the high-risk and low-risk groups. The features with $p<0.05$ were retained. Secondly, we used the Pearson correlation analysis to remove redundant features. For the feature pairs with absolute value of Pearson correlation coefficient greater than 0.9, features with larger mean absolute correlation were removed. After removing the irrelevant or redundant features, the least absolute shrinkage and selection operator (LASSO) was performed to determine the final features. The

LASSO method was designed to minimize the objective function. While the λ was larger, the coefficients of radiomics features were smaller. Features with nonzero coefficients were selected. The λ resulting the least mean difference between the predicted and actual result in the cross-validations was used to select the final features. To avoid multicollinearity and overfitting phenomena, LASSO algorithm was used to select the optimized subset of features and evaluate the corresponding coefficients with tenfold cross -validation.
